# Identification of the role of endoplasmic reticulum stress genes in endometrial cancer and their association with tumor immunity

**DOI:** 10.1186/s12920-023-01679-5

**Published:** 2023-10-25

**Authors:** Tang ansu Zhang, Qian Zhang, Jun Zhang, Rong Zhao, Rui Shi, Sitian Wei, Shuangge Liu, Qi Zhang, Hongbo Wang

**Affiliations:** 1 Department of Obstetrics and Gynecology, Union Hospital, Tongji Medical College, Huazhong University of Science and Technology, Wuhan, 430022 Hubei China; 2 Clinical Research Center of Cancer Immunotherapy, Wuhan, 430022 Hubei China

**Keywords:** Endometrial cancer, Endoplasmic reticulum stress, Bioinformatic analysis, Risk signature, Prognosis, Immune infiltration

## Abstract

**Background:**

Endometrial cancer (EC) is one of the worldwide gynecological malignancies. Endoplasmic reticulum (ER) stress is the cellular homeostasis disturbance that participates in cancer progression. However, the mechanisms of ER Stress on EC have not been fully elucidated.

**Method:**

The ER Stress-related genes were obtained from Gene Set Enrichment Analysis (GSEA) and GeneCards, and the RNA-seq and clinical data were downloaded from The Cancer Genome Atlas (TCGA). The risk signature was constructed by the Cox regression and the least absolute shrinkage and selection operator (LASSO) analysis. The significance of the risk signature and clinical factors were tested by time-dependent receiver operating characteristic (ROC) curves, and the selected were to build a nomogram. The immunity correlation was particularly analyzed, including the related immune cells, pathways, and immune checkpoints. Functional enrichment, potential chemotherapies, and in vitro validation were also conducted.

**Result:**

An ER Stress-based risk signature, consisting of TRIB3, CREB3L3, XBP1, and PPP1R15A was established. Patients were randomly divided into training and testing groups with 1:1 ratio for subsequent calculation and validation. Based on risk scores, high- and low-risk subgroups were classified, and low-risk subgroup demonstrated better prognosis. The Area Under Curve (AUC) demonstrated a reliable predictive capability of the risk signature. The majority of significantly different immune cells and pathways were enriched more in low-risk subgroup. Similarly, several typical immune checkpoints, expressed higher in low-risk subgroup. Patients of the two subgroups responded differently to chemotherapies.

**Conclusion:**

We established an ER Stress-based risk signature that could effectively predict EC patients’ prognosis and their immune correlation.

**Supplementary Information:**

The online version contains supplementary material available at 10.1186/s12920-023-01679-5.

## Introduction

EC is a worldwide gynecological malignancy, ranking as the fourth most common cancer and the sixth top cause of cancerous death in females. Although the median diagnostic age is already more than 61 years, EC still tends to occur in the younger population in recent decades [1]. The most significant risk factor for EC is exposure to estrogen, whether endogenous or exogenous, or even obesity. Other correlated factors include delayed menopause, early-onset menarche, nulliparity, diabetes, Lynch Syndrome, and Cowden Syndrome [2]. Despite many obvious achievements in EC treatment, such as surgery, radiotherapy, cytotoxic chemotherapy, and hormonal therapy [3], the morbidity and mortality rates of EC patients remain quite high globally. So, the action of identifying molecular mechanisms and then searching for novel treatment targets is still urgent for efficiently improving or even remarkably advancing the EC treatment.

As demonstrated, the tumoral abnormalities will cause the microenvironment mess, such as ischemia, hypoxia, oxidative stress, nutrient imbalance, and DNA damage, so as to disturb the homeostasis of endoplasmic reticulum, following misfolded or unfolded protein accumulation, thus leading to an intracellular state named ER Stress [4]. In turn, the continuous activation of the ER Stress will alter the tumorigenesis through both the transcriptional and translational pathways [5]. Furthermore, ER Stress can reprogramme the function of immune cells and thus apparently temper anti-cancer immunity [6]. ER Stress induces tumor cells to release factors that change the leucocytes around tumor cells, thus promoting tumor growth [7]. ER-stressed tumor cells secrete PD-L1 that drives M2 macrophage polarization, facilitating the escape from immune surveillance [8]. On the other hand, ER-stressed M1-like macrophages secrete DAMPs that lead to tumor cell death [9].

So far, continuously accumulated studies have presented that ER Stress should have played a critical role in endometrial carcinogenesis [10]. Protein and mRNA levels of several ER Stress-induced apoptosis indicators, including GRP78 and ATF6, are apparently increased in endometrial cancer immunohistochemical samples [11]. And GRP78 plasma membrane localization is elevated in endometrial cancer [12]. Therefore, cancer therapy focused on ER Stress is promising for deeper exploration.

In this paper, we constructed a bioinformatic method-based risk signature to explore the relevance and effect of ER Stress on EC progression. First, by combining ER Stress-related gene expression with clinical data, significantly differentially expressed genes between normal endometrial tissues and EC tissues were selected. After Cox analyses and LASSO regression analysis, 4 prognostic genes were filtered out. Based on these 4 genes that were identified as screening biomarkers of EC, a risk signature (risk score) was established by secondary clustering, which helped to discover molecular characteristics and immune infiltration specific to ER Stress of EC patients.

According to the survival prediction, EC patients with higher risk scores represented worse prognoses which indicated the clinical implication of our risk signature. For further analyses, results showed patients with higher risk scores were with less immune infiltration to evade the immune surveillance, as well as a lower rate of immune checkpoints, demonstrating that risk signature was capable to forecast the EC patients’ immunotherapies response. Furthermore, special potential chemotherapies were identified based on risk scores. 4 risk signature genes were then experimentally verified in EC cell lines and EC tissues. One challenge our risk signature face is that despite the strong correlation between immune and our risk signature, our risk signature was not significantly correlated with Tumor Mutation Burden (TMB), which is a potential biomarker in tumor immunotherapies selection [13, 14]. Overall, our study provided an effective prognostic signature based on ER Stress-related genes and helped personalized therapy for EC patients.

## Materials and methods

### Normal and tumor datasets

The transcriptome RNA-seq data of the EC samples (554 tumor datasets) and the normal samples (23 normal datasets) were obtained from TCGA database (https://portal.gdc.cancer.gov/repository) on 1st January 2023. The clinical features of EC patients were also obtained from the TCGA.

### Identification of ER stress-related differentially expressed genes

304 ER Stress-related genes (ERGs) were obtained from GSEA (http://www.gsea-msigdb.org/gsea/index.jsp), while 818 ERGs from GeneCards (http://www.genecards.org/) and 178 ERGS were finally selected after finishing intersection. The differentially expressed genes (DEGs) were selected based on the mRNA expression difference between the EC patients and the control via “limma” R package [15], and then following |log_2_fold change (FC)|>1 and false discovery rate (FDR) < 0.05. The Protein-Protein Interaction (PPI) networking was conducted by utilizing search tools for the retrieval of interacting genes (STRING) (http://string-db.org/) [16].

### Establishment and validation of the risk signature based on differentially expressed genes

To improve the predictive value of the DEGs in the EC patients selected, we already combined the DEGs with survival data from TCGA. The univariate Cox (uni-Cox) regression analysis was performed to filter prognostic ERGs from the DEGs with *P* < 0.05. 6 ERGs were identified for subsequent analysis. LASSO and the multivariate Cox (multi-Cox) regression analyses [17] were applied to establish a prognostic signature with “glmnet” R package [18]. 4 ERGs were eventually selected to calculate the risk score. The formula for performing risk score [19, 20] is presented below:

$$\text{r}\text{i}\text{s}\text{k} \text{s}\ \text{c}\text{o}\text{r}\text{e}={\sum }_{k=1}^{n}\text{e}\text{x}\text{p}\text{r} \left(\text{E}\text{R}\text{G}\text{s}\right)\times \text{c}\text{o}\text{e}\text{f} \left(\text{E}\text{R}\text{G}\text{s}\right)$$

where the expr is an expression of the ERGs, and the coef is the corresponding regression coefficient calculated by multi-variant Cox regression analysis.

Next, we summarized 523 samples and then randomly divided them into a training group and a testing group with a 1:1 ratio, and the extra portion was placed in the training group. The training group was mainly used to establish a risk signature, while the testing one was then utilized to validate the risk signature. The samples from both the training and testing groups were respectively separated into a high-risk subgroup and a low-risk subgroup according to the median risk score. The Kaplan-Meier analysis was performed on both the functioned groups above and ROC curves of the overall survival (OS) were employed to assess both the sensitivity and effectiveness of the risk signature. The patients with missing OS value or its value < 30 days were eliminated to reduce the statistical bias during comparison.

### Independent prognostic factor analysis

We tested whether the clinical characteristics (age, stage, grade) and the risk score could be used as independent prognostic factors through uni-Cox and multi-Cox regression analyses. By using “timeROC” and “survminer” R packages, both the time-dependent ROC curves and AUC were respectively constructed for comparing the efficiency of different predictive factors and the time-dependent survival rate.

### Function prediction analysis

To identify the possible functions of the ERGs chosen, we conducted both the gene ontology (GO) and the Kyoto Encyclopedia of Genes and Genomes (KEGG) [21–23] enrichment analyses based on the DEGs between the high- and low-risk subgroups by applying “clusterProfiler” R package [24]. The KEGG analysis was employed to find out the significantly enriched pathways, and then GO analysis consists of three enrichment parts: cellular component (CC), molecular function (MF), and biological process (BP), respectively.

### Nomogram and calibration analysis

The essential issues including the nomogram, the integrating risk score, age, stage, and grade along with a consistency index, were created to illustrate the prediction efficiency of 1-, 2-, and 3-year OS using the “rms” R package. The calibration curves were used to visualize the results and then evaluate the prediction consistency of the nomogram. The diagonal line (45°) can be recognized according to the best prediction value.

### Tumor immune analysis

We integrated the risk scores and the immune factors for predicting the relationship between the tumor immune and the ERGs. The immune cells and the immune pathway infiltrating scores were calculated according to the TCGA data via “gsva” R package [25]. The expression and survival differences of the high- and low-score patients in each immune cell/pathway type were further explored. A contrast was conducted according to the median immune cell or immune pathway enrichment level. The immune cell composition in the subgroups was analyzed carefully through the Cell type Identification By Estimating Relative Subsets of RNA Transcripts (CIBSORT) algorithm (http://cibersort.stanford.edu) with “corrplot” R package. The relationship between immune cell infiltration and ERGs expression was explored based on TIMER database (Tumor Immune Estimation Resource) [26], as well as the gene copy number variation (CNVs) of ERGs. The analyzed immune cells include B cells, CD4 + T cells, CD8 + T cells, macrophages, neutrophils, and dendritic cells.

### Potential treatment compounds

To find out a suitable potential clinical treatment that could be used for EC patients, we used the Genomics of Drug Sensitivity in Cancer (GDSC) (https://www.cancerrxgene.org/) database to calculate the half-maximal inhibitory concentration (IC50) of the compounds for predicting the sensitivity via “pRRophetic” R package [27].

### Verification of target ERGs in databases

The protein expression in EC tissues and normal tissues was verified according to the HPA (The Human Protein Atlas) [28]. And the mRNA expression was examined from the CCLE (Cancer Cell Line Encyclopedia).

### Quantitative real-time polymerase chain reaction

A quantitative Real-Time Polymerase Chain Reaction (qRT-PCR) was used to verify the expression of the target ERGs in EC cell lines. The total RNA was extracted from the EC cells with TRIZOL reagent (Takara, Otsu, Japan) according to the protocol, and then a 20 µl corresponding cDNA was reversely transcribed with Hiscript@ QRT 157 SuperMix (Vazyme, Nanjing, China). The PCR reaction steps were then followed as different configurations: 95 °C for 30 s, 95 °C for 5 s, and 60 °C for 1 min with 40 cycles. The qRT-PCR process was performed through the CFX Connect Real-Time PCR Detection System (Bio-Rad, 161 Hercules, CA, USA) with SYBR green supermix (Vazyme, Nanjing, China). β-Actin acted as an endogenous control to normalize the expression of each target ERG, and the relative expression levels were calculated with the 2^−ΔΔCt^ method (ΔCt = ΔCt target – ΔCt β-actin). The primer sequences were already listed in Table 1.

**Table 1:** The primers sequence of 4 prognostic-related ERGs

Gene	Primer Sequences
TRIB3-F	TGCGTGATCTCAAGCTGTGT
TRIB3-R	GCTTGTCCCACAGGGAATCA
CREB3L3-F	ATCTCCTGTTTGACCGGCAG
CREB3L3-R	GTCGTCAGAGTCGGGGTTTG
XBP1-F	AGGAGTTAAGACAGCGCTTGGGGATGGAT
XBP1-R	CTGAATCTGAAGAGTCAATACCGCCAGAAT
PPP1R15A-F	CTGGCTGGTGGAAGCAGTAA
PPP1R15A-R	TATGGGGGATTGCCAGAGGA

### Statistical analysis

The statistical analyses were performed with R in version 4.1.0. The classification of variables in the training and testing groups was decided by the Person chi-square test. The overall survival was analyzed via Kaplan–Meier method along with log-rank test. An independent prognostic analysis was conducted by uni- and multi-Cox regressions. The analyses of several typical characteristics such as the clinicopathological factors, the risk score, and the immune infiltration levels, were assessed using the Wilcoxon test. In general, *P* < 0.05 was considered as being significantly different.

## Results

### Identifying 4 prognostic-related differentially expressed ER stress genes

**Fig. 1:**
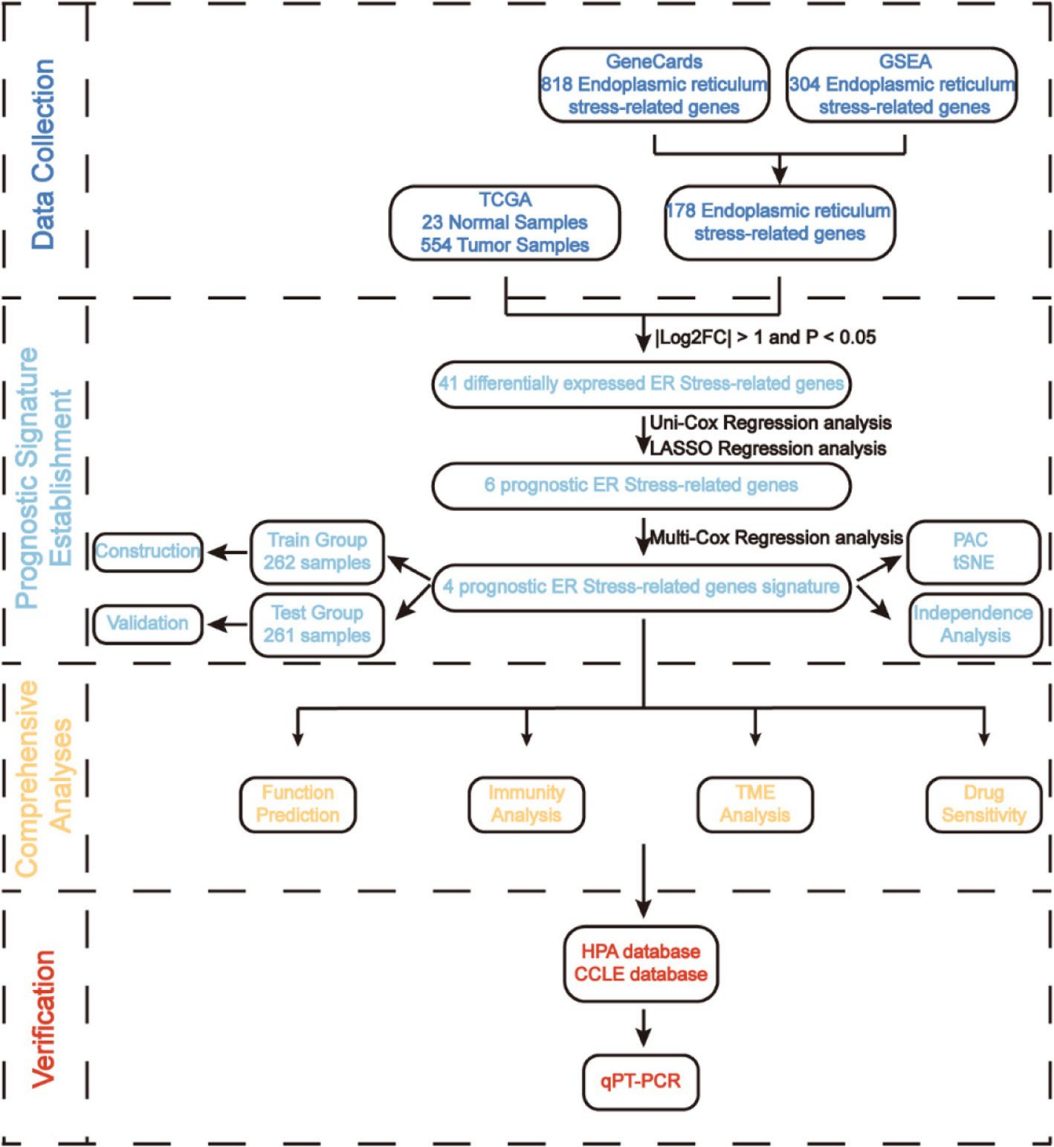
Main flowchart of the working procedure including an initial data collection, a key prognostic signature establishment, several comprehensive analyses, and a final verification

**Fig. 2:**
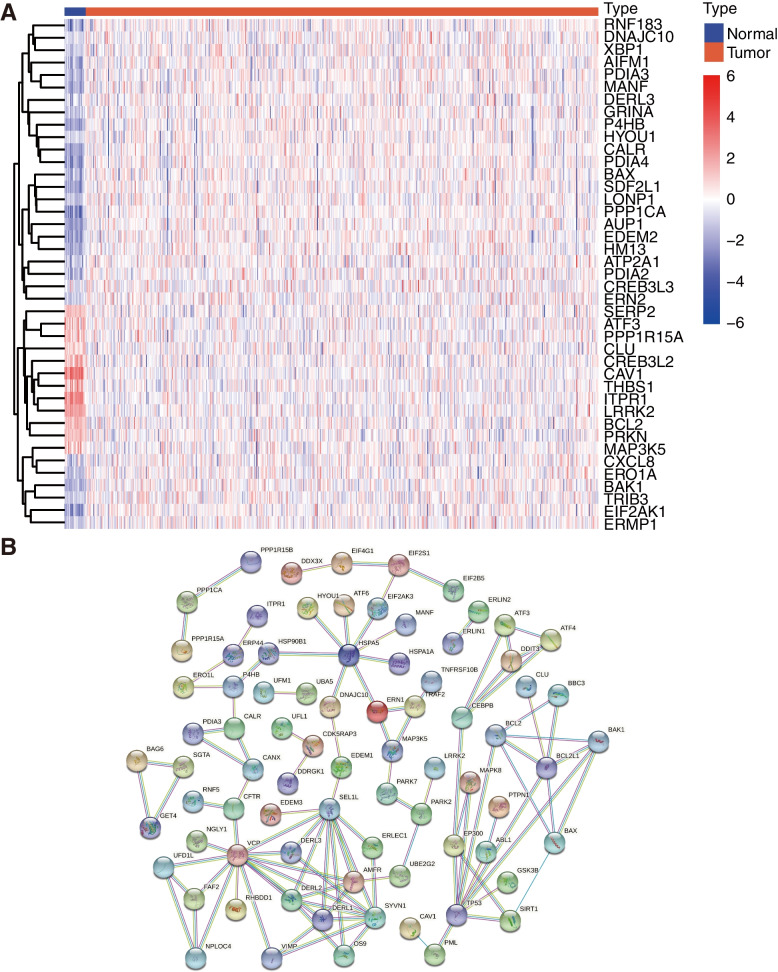
Expression of DEGs between the case of EC and the normal patients of TCGA corhort. **A** Heatmap shows DEGs between tumor (red) and normal (blue) samples. **B** PPI network indicates the interaction between DEGs

The process of this study was exhibited in the flow chart (Fig. 1). We already obtained 23 normal samples and 554 tumor samples with mRNA expression, and the clinical data from TCGA in total. 178 ER Stress-related gene expression was compared practically between the normal and tumor groups, and then 41 ERGs were selected as differentially expressed genes based on |log_2_FC|>1 and FDR < 0.05, among them, 12 samples (CAV1, ITPR1, LRRK2, THBS1, ATF3, BCL2, SERP2, MAP3K5, CLU, PRKN, CREB3L2, PPP1R15A) were downregulated, while 29 samples (LONP1, GRINA, EIF2AK1, HYOU1, AUP1, BAX, EDEM2, AIFM1, HM13, PDIA3, ERMP1, DNAJC10, XBP1, CXCL8, ATP2A1, MANF, DERL3, CALR, BAK1, ERO1A, PPP1CA, PDIA4, CREB3L3, P4HB, SDF2L1, TRIB3, RNF183, PDIA2, ERN2), were upregulated. The expression level of the 41 DEGs was presented in the heatmap (Fig. 2A). We further conducted the PPI manipulation (Fig. 2B), which presented the direct interaction between the proteins encoded by DEGs to better understand the potential roles of ER Stress in manipulating EC progression. 132 ERGs were identified as hub genes with a restriction of the interaction score as the highest confidence of 0.9, among them 32 were DEGs (Table 2).

**Table 2:** List of 32 DEGs with 0.9 interaction score between EC tissue and normal endometrial tissue in TCGA

AIFM1	CLU	ERN2	PDIA3
ATF3	CREB3L2	HM13	PDIA4
AUP1	CREB3L3	HYOU1	PPP1CA
BAK1	CXCL8	ITPR1	PPP1R15A
BAX	DERL3	LRRK2	SDF2L1
BCL2	DNAJC10	MANF	THBS1
CALR	EDEM2	MAP3K5	TRIB3
CAV1	EIF2AK1	P4HB	XBP1

### Construction and validation of the 4 ERGs-based risk signature for prognostic effectiveness

**Fig. 3:**
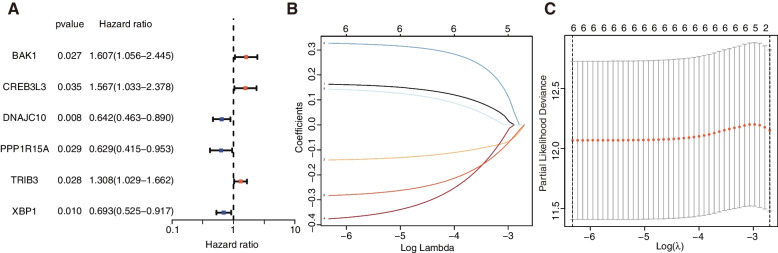
Construction and validation of the risk signature based on ERGs. **A** Uni-Cox regression analysis of 6 ERGs with *P* < 0.05. **B** LASSO regression analysis of 6 ERGs. **C** Cross-validation of LASSO regression analysis

To identify the candidates for constructing a risk signature, we first filtered 6 genes related to the prognosis of EC according to the uni-Cox regression analysis with *P* < 0.05 (BAK1, CREB3L3, DNAJC10, PPP1R15A, TRIB3, XBP1) (Fig. 3A). Then, the LASSO regression analysis confirmed that these 6 genes are related to the survival of the EC patients (Fig. 3B, C). To avoid overfitting the prognostic signature, we applied the minimum likelihood of deviance of the first-rank value of Log(λ) during LASSO regression analysis. We also performed a multi-Cox regression analysis, and then 4 genes were finally identified as significant risk signature genes (CREB3L3, PPP1R15A, TRIB3, XBP1). Among them, PPP1R15A and XBP1 are related to the protective effect with hazard ratio (HR) < 1, whereas CREB3L3 and TRIB3 are the increased risk factors with HR > 1. The calculation formula of the risk scoring is as below:

$$\mathrm{Risk}\;\mathrm{Score}\;=\;(0.3965)\times(\mathrm{CREB}3\mathrm L3\;\exp.)+(-0.4152)\times(\mathrm{PPP}1\mathrm R15\mathrm A\;\exp.)+(0.2223)\times(\mathrm{TRIB}3\;\exp.)+(-0.3508)\times(\mathrm{XBP}1\;\exp.)$$

**Fig. 4:**
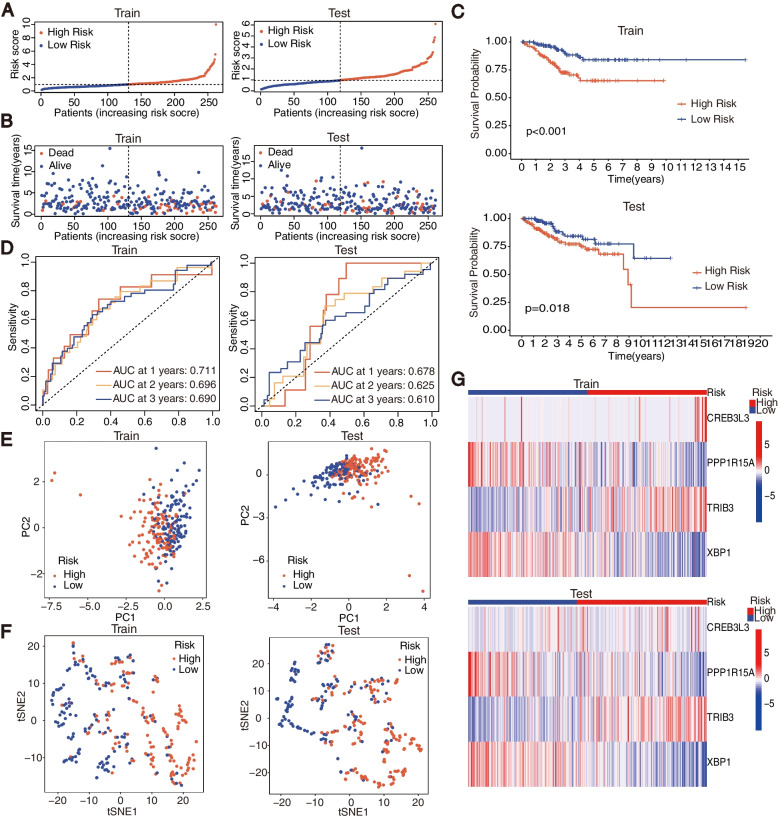
Survival analysis of the training and testing cohorts from TCGA. **A** Risk Score, (**B**) Survival State, (**C**) Kaplan-meier survival, (**D**) ROC survival, (**E**) PCA plot, (**F**) tSNE plot, (**G**) expression heatmap of risk signature

**Fig. 5:**
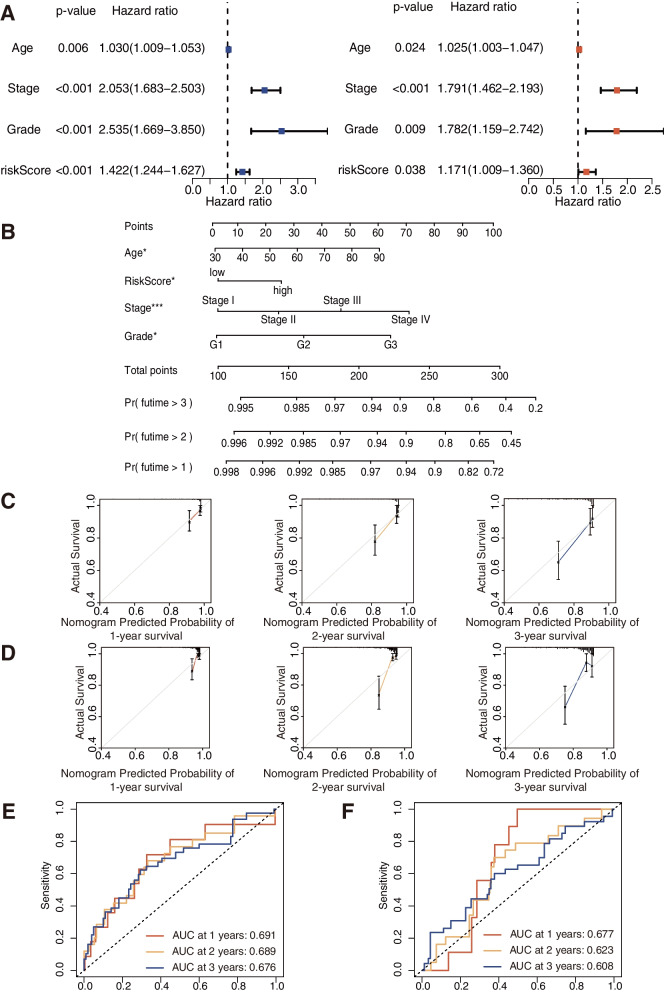
Risk score is utilized as an independent predictor for different issues. **A** Uni-Cox and Multi-Cox regression analysis of risk score and clinical characteristics. **B** Establishment of prognostic nomograph to predict survival of EC. **C, D** Calibration curves of the nomogram to predict 1-year and 2-year and 3-year survival in the training/testing group. **E, F** ROC curves of nomograph to predict 1-year and 2-year and 3-year prognostic value in the training/testing group

After screening out, we first sorted out 523 clinical samples from TCGA. Next, we randomly divided the patients into the training and testing groups with 1:1 ratio (of 262 samples in the training group and 261 samples in the testing group). The risk scores of the training and testing groups were then calculated separately. Based on the median score, all samples from both the training and testing groups were classified into high- and low-risk subgroups. As demonstrated, patients from the low-risk subgroup already exhibit a better prognosis with statistically lower risk and fewer deaths than the high-risk subgroup (Fig. 4A, B). Furthermore, the OS between the high- and low-risk subgroups were significantly different with *P* < 0.05 (Fig. 4C). The AUCs of the training and testing cohorts were 0.711 or 0.678 at 1 year, 0.696 or 0.625 at 2 years, 0.690 or 0.610 at 3 years, respectively (Fig. 4D). The above data imply that the risk score can be used as a valid factor for effectively predicting the prognosis of EC patients. Both the PCA and tSNE analyses indicate that the risk signature constructed by us can be utilized to distinguish high-risk patients from low-risk patients (Fig. 4E, F). The heatmaps constructed by us can also be used to visualize the variance of the prognostic ERGs expression between the high- and low-risk subgroups (Fig. 4G). It should be noted that the risk score can be used as an independent predictor from uni-Cox and multi-Cox regression analyses that the HR of the risk score and 95% confidence interval (95% CI) was 1.422, and 1.244–1.627 (*P* < 0.05) in uni-cox regression while 1.717, and 1.009–1.360 (*P* < 0.05) in multi-cox regression (Fig. 5A).

### Nomograph construction and the clinical correlation

According to 4 independent prognostic factors such as risk score, age, stage, and grade (all *p* < 0.05 in uni-Cox and multi-Cox), we built a clinical nomogram to predict the prognosis of the EC patients through a comprehensive score (Fig. 5B). We also utilized the 1- and 2- and 3-year calibration plots to present or prove that the nomogram had a reliable prediction efficiency, which can be used to exhibit excellent accordance between the predictive and actual survival in both the training and testing groups (Fig. 5C, D). As shown, the diagonal line presents the ideal prediction. Furthermore, the performed ROC analyses show that the AUC of the 1-and 2- and 3-year predictive survival according to the nomograph are 0.691and 0.689and 0.676 in the training group, and then 0.677 and 0.623 and 0.608 in the testing group, which means that the nomograph can be effectively used to predict (Fig. 5E, F).

### Enriched functions based on the risk signature

**Fig. 6:**
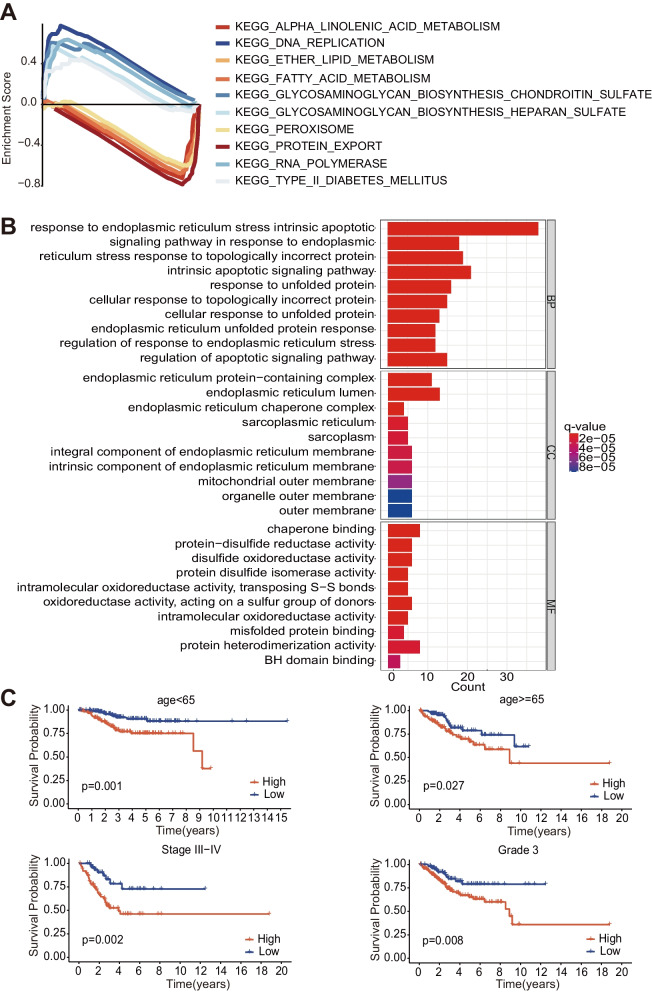
Functional enrichment analysis about the ERGs between the high-risk and the low-risk subgroups. **A** KEGG pathway enrichment of ERGs are present. **B** GO enrichment analysis of ERGs. **C** Kaplan-meier survival analysis of clinical characteristics

To further illustrate the biological functions and the enriched pathways of the risk signature, we performed both the KEGG pathway analysis and the GO enrichment analysis between the high- and low-risk subgroups. The KEGG demonstrates that the pathways enriched in the low-risk subgroup are: protein export, alpha-linolenic acid metabolism, fatty acid metabolism, ether lipid metabolism, and peroxisome, respectively, whereas the pathways in the high-risk subgroup are: DNA replication, RNA polymerase, type II diabetes mellitus, glycosaminoglycan biosynthesis chondroitin sulfate and heparan sulfate (Fig. 6A). Moreover, the GO showed that genes are mainly enriched in the response to the factors such as endoplasmic reticulum stress, endoplasmic reticulum lumen, and endoplasmic reticulum protein-containing complex, which indicates that the genes chosen by us are closely related to the ER Stress to some extent (Fig. 6B).

### Relationship between 4 ERGs-based risk signature and clinical characteristics

The correlation between the survival probability and different clinicopathological characteristics was further interrogated. The data demonstrate that the patients from the low-risk subgroup present much better survival overall, and the risk scores are significantly related to the issues such as age, grade, and stage features (Table 3). The patients with a higher stage and grade level are more likely to present significant differences based on the risk score (Fig. 6C). Instructively, the risk signature constructed by us is convictive and reliable for diagnosing and treating EC patients.

**Table 3:** Endometrial cancer patient clinical characteristics from TCGA

Variables	Number of cases	Percentage(%)
Age(year) < 65/≥65	288/247	53.8/46.2
Grade G1/G2/G3	99/1212/316	18.4/22.7/58.8
Stage I-II/III-IV	390/147	72.6/27.4

### Immune infiltration differences between two subgroups

**Fig. 7:**
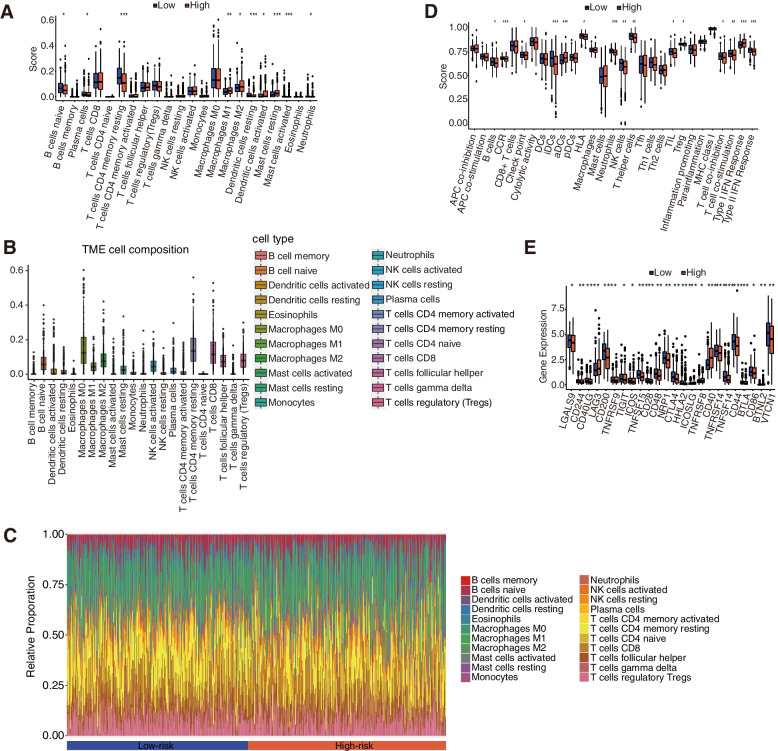
Immune cell infiltration and immune pathways analysis based on the risk score. **A** ssGSEA score of immune cells in high- and low-risk subgroups. **B** CIBERSORT analysis of immune cell composition. **C** CIBERSORT analysis of immune cell relative percentage in high- and low-risk subgroups. **D** ssGSEA score of immune pathways in both the high- and the low-risk subgroups. **E** Immune checkpoints analysis in high- and low-risk subgroups. *P < 0.05, **P < 0.01, ***P < 0.001

**Fig. 8:**
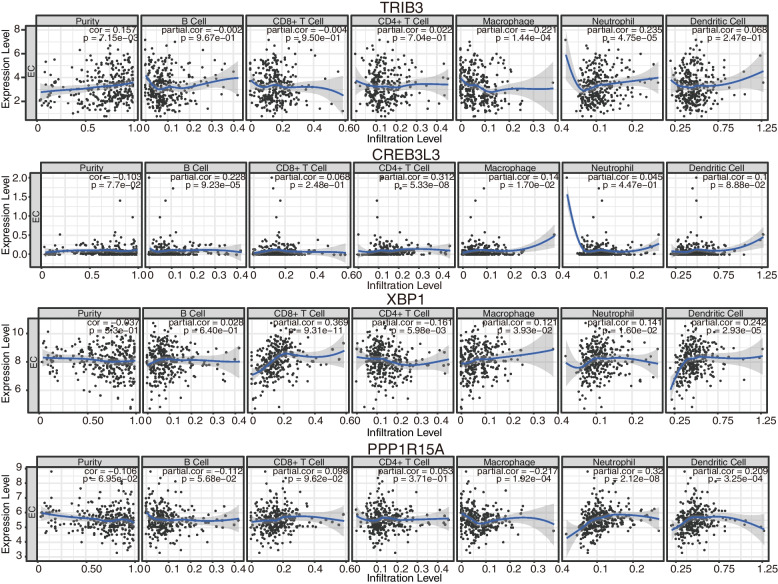
The relationship analyses of immune cell infiltration level and 4 ERGs mRNA expression based on TIMER database

**Fig. 9:**
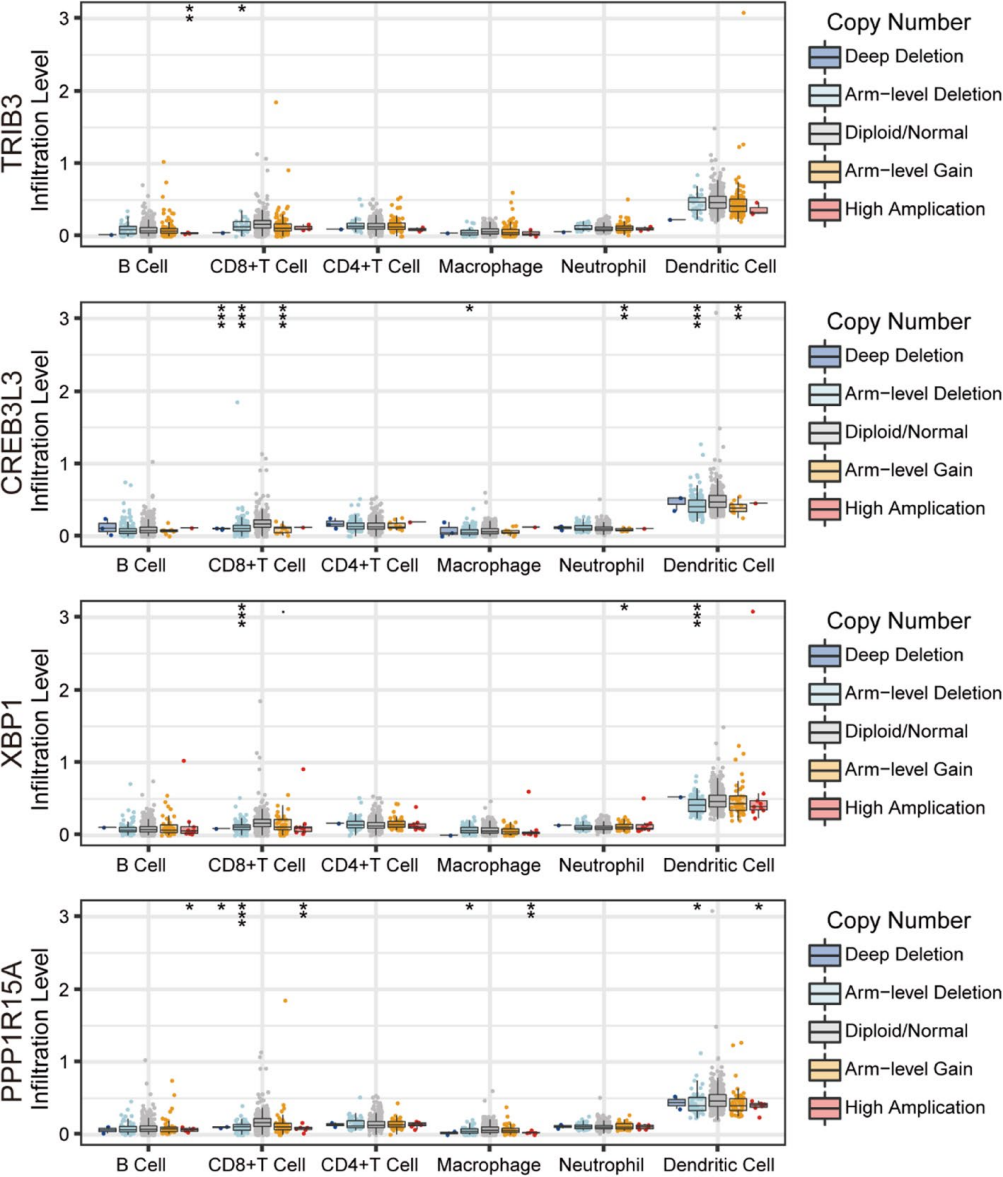
The relationship analyses of immune cell infiltration level and 4 ERGs CNVs based on TIMER database. *P < 0.05, **P < 0.01, ***P < 0.001

Generally, immune abnormalities will play a critical role in oncogenesis, thus we continuously analyzed the enrichment of the immune cells and then immune pathways based on the risk score through a single sample gene set enrichment analysis (ssGSEA) method. According to the box plot, cells such as B cells naïve, plasma cells, T cells CD4 memory resting, macrophages M1, macrophages M2, dendritic cells resting, dendritic cells activated, mast cells resting, mast cells activated, and neutrophils, are all sensitive to the risk score with *p* < 0.05 criteria. Among them, only macrophages M1 and macrophages M2 are higher in the high-risk subgroup (Fig. 7A). Continuously, we analyzed the survival probability of the samples including immune cells, dendritic cells activated, NK cells activated, plasma cells, T cells CD8, and T cells regulatory (Tregs), which all exhibited a significant difference between the high- and low-risk subgroups (Fig. S1A). The dendritic cells were activated and the plasma cells showed a better prognosis in the low-risk subgroup, whereas the NK cells were activated, and the T cells CD8 and T cells regulatory (Tregs) were in the high-risk subgroup. It is evident that the risk signature was closely related to immune cell infiltration, thus we performed the CIBERSORT to further explore the tumor microenvironment (TME). The compositions of 22 immune cells in the samples and the relative percentage filtered in the high- and low-risk subgroups were analyzed further (Fig. 7B, C). The high-risk subgroup had a higher proportion of anti-inflammatory macrophage M2 cells, while the low-risk subgroup had an increased proportion of resting memory CD4 T cells, activated dendritic cells and mast cells, indicating the potential anti-tumor effects of the low-risk subgroup. Meanwhile, we further investigate the correlation between the abundance of several typical immune cells and the mRNA expression of 4 ERGs through TIMER database (Fig. 8). Macrophages are significantly related to all 4 ERGs. TRIB3 expression level is only correlated with the abundance of another immune cell, neutrophil. CREB3L3 is strongly correlated with B cells and CD4^+^ T cells with similarly positive tendencies. Except for CD4 + T cells, XBP1 shows significantly negative correlations with CD8 + T cells, neutrophils, and dendritic cells. PPP1R15A is positively related to the abundance of neutrophils and dendritic cells. Compared with the diploid/normal group, we further figure out that immune cell infiltration is also related to CNVs of 4 ERGs (Fig. 9). Arm-level deletion of TRIB3, XBP1, and PPP1R15A decreases the infiltration of immune cells. Compared with the arm-level deletion, the arm-level gain of CREB3L3 is more likely to decrease the infiltration of CD8^+^ T cells, neutrophils, and dendritic cells. High amplification of PPP1R15A mainly affects B cells, CD8^+^ T cells, macrophages, and dendritic cells. TRIB3, CREB3L3, and XBP1 are lack of deep deletion.

Besides the immune cells, we have also explored the relationship between the immune pathways and the risk score. The box plot also shows the typical characteristics of the microstructures including B_cells, CCR, checkpoint, iDCs, aDCs, HLA, neutrophils, NK_cells, T_helper_cells, TIL, Treg, T_cell_co-inhibition, T_cell_co-stimulation, Type_I_IFN_Response, and Type_II_IFN_Response, are significantly different according to the risk score (Fig. 7D). The survival analysis of the immune pathways was further conducted, and the results indicate that the immune pathways with prominent survival differences, all have better survival in the high-risk subgroups than in the low-risk subgroup (Fig. S1B). Because the immune checkpoints are significantly different between the two subgroups, the expression distinction was then analyzed, where only a few are expressed higher in the high-risk subgroup, like CD 40. As demonstrated, the majority of the immune checkpoints are higher in the low-risk subgroup, especially corresponding to CTLA4 and CD28. Unfortunately, there is no significant difference in PD-1 or PD-L1 (Fig. 7E).

The above outcomes suggest that the risk score will exhibit a considerable impact on the prognosis of various immune cells and pathways, which may helpfully predict new individualized immunotherapy for EC patients.

### Consensus clusters selection based on the expression level

**Fig. 10:**
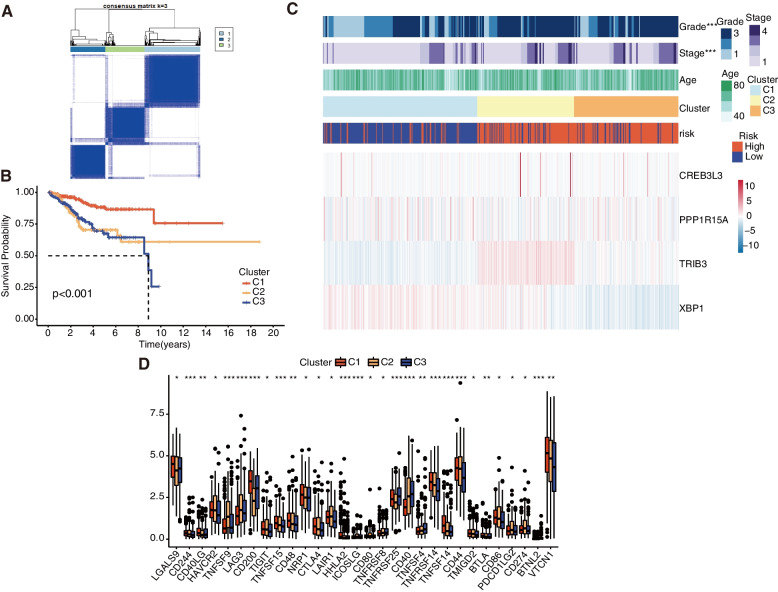
Classification of the EC samples based on ERGs. **A** The EC patients were divided into 3 clusters based on the consensus matrix (k = 3). **B** Kaplan-meier survival analysis based on 3 clusters. **C** Heatmap of 3 clusters and clinical characteristics. **D** Immune checkpoints enrichment analysis based on 3 clusters. *P < 0.05, **P < 0.01, ***P < 0.001

The consensus clustering analysis of all 523 EC samples in the TCGA cohort for recognizing the relationship between the expression levels of 4 risk-signature ERGs and subtypes, was performed. We already selected the clustering variable (*k*) = 3 from 2 to 9 based on a higher intragroup correlation and a lower intergroup correlation (Fig. 10A), with 225 cases in cluster 1, 142 cases in cluster 2, and 156 cases in cluster 3. Survival analysis indicates that there exists an evident difference in survival among the three subgroups above. As compared to Cluster 2 and Cluster 3, Cluster 1 has a much better prognosis (Fig. 10B).

The issues including the information on mRNA expression, clusters, and clinical characteristics concerning grade and stage, and age, were integrated and displayed in the heatmap. As shown, the parameter grade and stage were significantly different among the three clusters (Fig. 10C). Furthermore, we further explored the expression differences of 30 immune checkpoints in three clusters, among them, the samples of CD244, TNFSF9, Lag3, CD200, TNFSF15, HHLA2, ICOSLG, TNFRSF25, CD40, TNFRSF14, TNFSF14, CD44, and BTNL2, are more statistically significant than others (*p* < 0.001) (Fig. 10D).

### Diverse potential chemotherapies of two subgroups

**Fig. 11:**
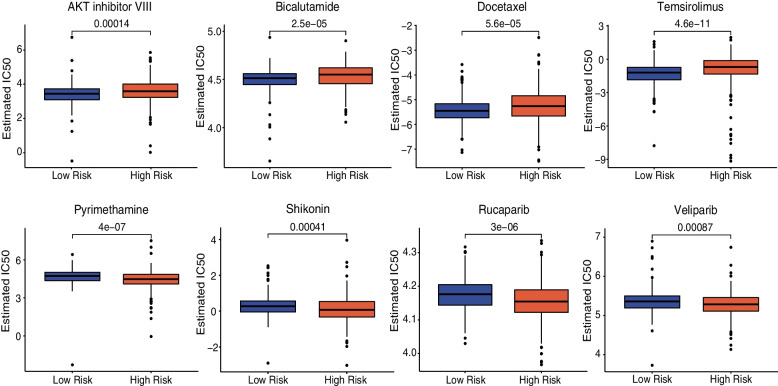
Potential chemotherapy and immunotherapy sensitivity prediction between the low-risk and the high-risk subgroups

Because chemotherapy is one of the effective and widely-accepted treatments for EC patients, we further did a prediction of the sensitivity of potential chemotherapies based on IC50 for each sample in both the low- and high-risk subsgroups. Generally, the patients with a lower risk score are more sensitive to medicines such as AKT inhibitor VIII, Bicalutamide, Docetaxel, and Temsirolimus. While the higher-risk score patients should be more sensitive to Pyrimethamine, Shikonin, Rucaparib, and Veliparib (Fig. 11). These findings might provide new options for future clinical treatment.

#### Dual verification of the 4 ERGs-based risk signature

**Fig. 12:**
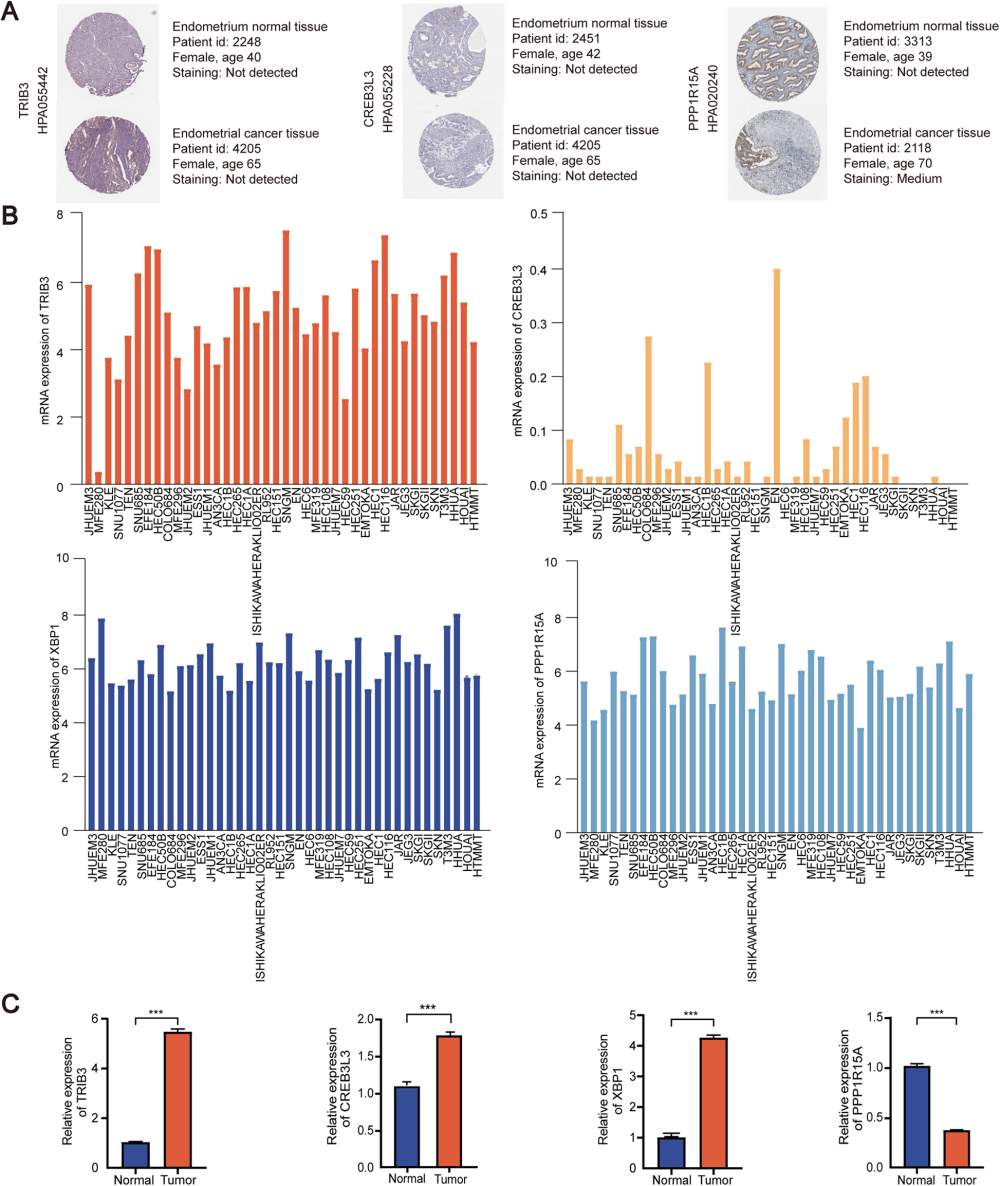
Verification of gene expression. **A** The protein expression level from HPA database. **B** mRNA expression level of EC cell lines from CCLE database. **C** Gene expression with clinical tissues. ***P < 0.001

The protein level of 4 risk-signature ERGs (CREB3L3, PPP1R15A, TRIB3, XBP1) was verified according to the HPA database (Fig. 12A). Unfortunately, we couldn’t obtain successfully a protein expression of XBP1 in the HPA database. Besides this gene, we find that both the CREB3L3 and TRIB3 are upregulated, but PPP1R15A is downregulated on the protein level. The mRNA levels of 4 ERGs are different in 40 EC cell lines (Fig. 12B).

We then performed qRT-PCR to verify the mRNA expression of 4 risk-signature ERGs (CREB3L3, PPP1R15A, TRIB3, XBP1) in EC and normal tissues, separately. The expression level of these genes differs significantly between the tumor and normal tissues, but CREB3L3 and TRIB3, and XBP1 are overexpressed, while PPP1R15A is expressed conversely (Fig. 12C). The primer sequences are provided in Table 1. Overall, the experimental results further confirm the reliability of the risk signature (*p < 0.05, **p < 0.01, and ***p < 0.001).

## Discussion

EC is one of the highly heterogeneous malignancies developed in the female genital tract. A great number of studies have reported that endoplasmic reticulum stress is closely related to the progression and outcome of different types of cancer, including the EC [11, 29], but the mechanisms have not yet been fully discovered. Furthermore, most studies investigated the impact of ER Stress on cancer biological activities, just a few focused on the prognostic significance of ER Stress-related genes, particularly in the case of EC. Therefore, it is necessary to develop a predictive risk signature based on the relationship between both EC and ER Stress. Here, we have built a stable prognostic signature to predict the prognostic ER Stress-related genes and then divided them into different subgroups, to systemically learn the relationship among genes, tumor microenvironment, and immune activities. It should be noted that we further predict the potential chemotherapies that may be effectively utilized in future clinical treatment.

First of all, we successfully constructed a reliable 4-gene ER Stress-related prognostic signature for EC with TCGA gene expression and clinical files. 4 prognostic ERGs (CREB3L3, PPP1R15A, TRIB3, and XBP1) were screened from 178 ER Stress-related genes via Cox and LASSO regression analyses. Two subgroups were classified based on the risk score. A nomograph was employed to prove the reliable predictive value of the risk signature. OS results suggested a strong prognostic correlation between EC and ER Stress. The prediction ability of the risk signature was obviously affected by the clinical features that patients with lower risk scores presented better prognoses.

The functional enrichment highlighted the interactive mechanisms between ER Stress and endometrial carcinogenesis. GO enrichment suggested that ERGs are mainly involved in apoptotic regulation besides ER Stress related to signaling pathways. The mutual effect between ER Stress and apoptosis in driving cancers has been well explained [30]. KEGG enrichment presenting pathways enriched in the high-risk subgroup show more carcinogenesis correlation, like DNA replication. The metabolism-related pathways are highly enriched in the low-risk subgroup, such as fatty acid, protein, and RNA metabolism, to confirm that the patients from the high-risk subgroup exhibit unsatisfactory prognoses and immune-suppressive status.

Considering the previous studies have demonstrated the critical role of immune-related activities in endometrial cancer progression, we further analyze the differences in immune infiltration between the subgroups. From ssGSEA results, B cells naïve, plasma cells, and resting memory CD4^+^ T cells are evidently downregulated in the high-risk subgroup. Both naïve B cells and plasma cells are critical subpopulations of B cells, naïve B cells are the precursor of functional B cells and plasma cells are recognized as the basis of humoral immunity [31, 32]. B cells and plasma cells are associated with better survival, and particularly B-cell markers can prolong survival specifically in high-grade tumors [33]. Memory T cells play important roles in T cell persistence and tumor immunotherapy efficacy [34]. Thus, this observation may demonstrate the higher chance of the high-risk subgroup to be a suppressive immune status than the lower-risk subgroup. By contrast, M1 and M2 macrophages and mast cells are upregulated in the high-risk subgroup, indicating their relationship with the unfavorable prognosis, which has been proved by previous studies [35].

CIBERSORT analysis demonstrates the top one cell enriched in the low-risk subgroup with a significant difference is resting memory CD4 T cells, indicating the protective role in the EC progression. Similar to the ssGSEA result, the high-risk subgroup had a higher proportion of anti-inflammatory macrophage M2 cells. So far, the immune pathway analysis has paid more attention to CCR and DCs, and IFN responses. According to the differences in checkpoints, we further find that several crucial immune checkpoints, such as CTLA 4, and CD 28, are differentially expressed. The drug targeting immune checkpoints could be used alone or combined with other appropriate treatments, and thus present an advantage of lower toxicity and much higher effectiveness to some extent [36]. In general, ERGs will influence immune infiltration and prognosis. It should be noted that understanding the mechanisms is not enough for the EC patients’ treatment decision-making. Thus, the potential chemotherapies are continuously discussed. Patients in the high-risk subgroup are more sensitive to the components such as AKT inhibitor VIII, Bicalutamide, Docetaxel, and Temsirolimus, whereas the patients in the low-risk subgroup will benefit from the medications such as Pyrimethamine, Shikonin, Rucaparib, and Veliparib.

CREB3L3 (cAMP-responsive element-binding protein 3 like 3) is a membrane-bound transcription factor located in the ER and structurally similar to ATF6 that could be proteolytically activated by ER Stress [37]. CREB knockdown in macrophages could downregulate M2 marker genes, then improve insulin resistance, suggesting that CREB is important in maintaining insulin sensitivity in white adipose tissue via its initiation of the innate immune system [38].

PPP1R15A (protein phosphatase 1 regulatory subunit 15 A), also known as GADD34, is a stress-inducible eIF2α phosphatase, which can accelerate cell death by boosting protein synthesis and activating death-related pathways [39]. PPP1R15A has been proven to be a hypoxia/autophagy-related gene in breast cancer respectively [40, 41]. However, there are just a few studies focusing on the action of PPP1R15A in endometrial cancer progression to date, which requires more studies.

TRIB3 is one of the Tribbles Pseudokinase homologs with a weak ATP affinity. Despite the absence of kinase activity, TRIB3 is an adaptor/scaffold protein for numerous functional proteins [42]. TRIB3 promotes endometrial malignant actions via elevating CTNNB1 transcription [43]. And it’s found that TRIB3 expression change is part of the anti-endometrial cancer activities in one ongoing clinical trial [44].

XBP1 (X-box binding protein 1) belongs to the CREB/ATF basic leucine zipper (bZIP) protein family. XBP1 can be alternatively spliced into two isoforms, the unspliced isoform (XBP1-u) is spliced into the spliced isoform (XBP1-s) under ER Stress [45]. XBP1 is highly enriched in endometrioid endometrial tumors [46]. One recent study indicates that XBP1 potentially distinguishes the polymerase epsilon exonuclease (POLE) from the copy number (CN)-low endometrial cancer subtype [47]. Therefore, XBP1 may be a strong promoter of endometrial carcinogenesis. Otherwise, some studies suggest the dual roles of XBP1. XBP1-u inhibits autophagy [48] and protects cells from oxidative stress [49]. XBP1-s promotes cell death by altering calcium levels under strong ER Stress, while under non-fatal ER Stress, XBP1-s protects cell survival by transcriptionally regulating UPR target genes [45]. Together, it may explain that XBP1 is identified as the protective factor in our risk signature, but expressed higher in EC tissues than normal tissues.

Despite already presenting an obvious value of the risk signature developed by us, some limitations and deficiencies still exist. First off, the RNA-sequence and the sample data used by us are only acquired from TCGA, which might cause bias in the following calculations. It may be a better approach by combining the data from other resources to derive comprehensive results. Second, the specific mechanisms based on ERGs should be dug out through more experiments. Last but not least, as the patients from the high-risk subgroup present a relatively worse prognosis, closer follow-ups should be required to further prove the risk signature and nomograph viability.

## Conclusion

In this paper, an effective ER Stress-related risk signature based on 4 prognostic genes is successfully established by us. The risk signature can accurately predict the prognosis and help treatment-decision making for EC patients. The fundamental relationship between immunity, TME, and EC-specific ER Stress, is discussed carefully through the risk signature. We also verified the reliability of the risk signature with basic experiments.

### Supplementary Information

**Additional file 1: Fig. S1. **Further survival analysis linked with immune infiltration. A Kaplan-meier analysis of significantly different immune cells. B Kaplan-meieranalysis of significantly different immune pathways.

## Data Availability

All the data involved in this study were collected from open sources, TCGA (http://portal.gdc.cancer.gov), GDSC (http://www.cancerrxgene.org/), TIMER (https://cistrome.shinyapps.io/timer/), CCLE (http://www.broadinstitute.org/ccle), and HPA (http://www.proteinatlas.org/) databases.

## Abbreviations

EC: Endometrial cancer
ER Stress: endoplasmic reticulum stress
TCGA: The cancer genome atlas
GSEA: Gene set enrichment analysis
HPA: The human protein atlas
CCLE: Cancer Cell Line Encyclopedia
GDSC: The genomics of drug sensitivity in cancer
ERGs: ER Stress-related genes
DEGs: Differentially expressed genes
OS: Overall survival
ROC: Receiver operating characteristic
AUC: Area under the curve
PPI: Protein-protein interaction
KEGG: the Kyoto Encyclopedia of Genes and Genomes
GO: the Gene Ontology
CNVs: Copy Number Variation
IC50: Half-maximal inhibitory concentration
qRT-PCR: Real-time quantitative RT-PCR
CREB3L3: cAMP-responsive element-binding protein 3 like 3
PPP1R15A: Protein phosphatase 1 regulatory subunit 15 A
TRIB3: Tribbles pseudokinase 3
XBP1: X-box binding protein 1
