# Supplementary material for: Identification of the role of endoplasmic reticulum stress genes in endometrial cancer and their association with tumor immunity
Source: BMC Med Genomics. 2023 Oct 25;16:261. doi: 10.1186/s12920-023-01679-5 (PMC10599039; doi:10.1186/s12920-023-01679-5)
Supplement: Supplementary file 1 — Additional file 1: Fig. S1. Further survival analysis linked with immune infiltration. A Kaplan-meier analysis of significantly different immune cells. B Kaplan-meieranalysis of significantly different immune pathways. [file 12920_2023_1679_MOESM1_ESM.docx]

**
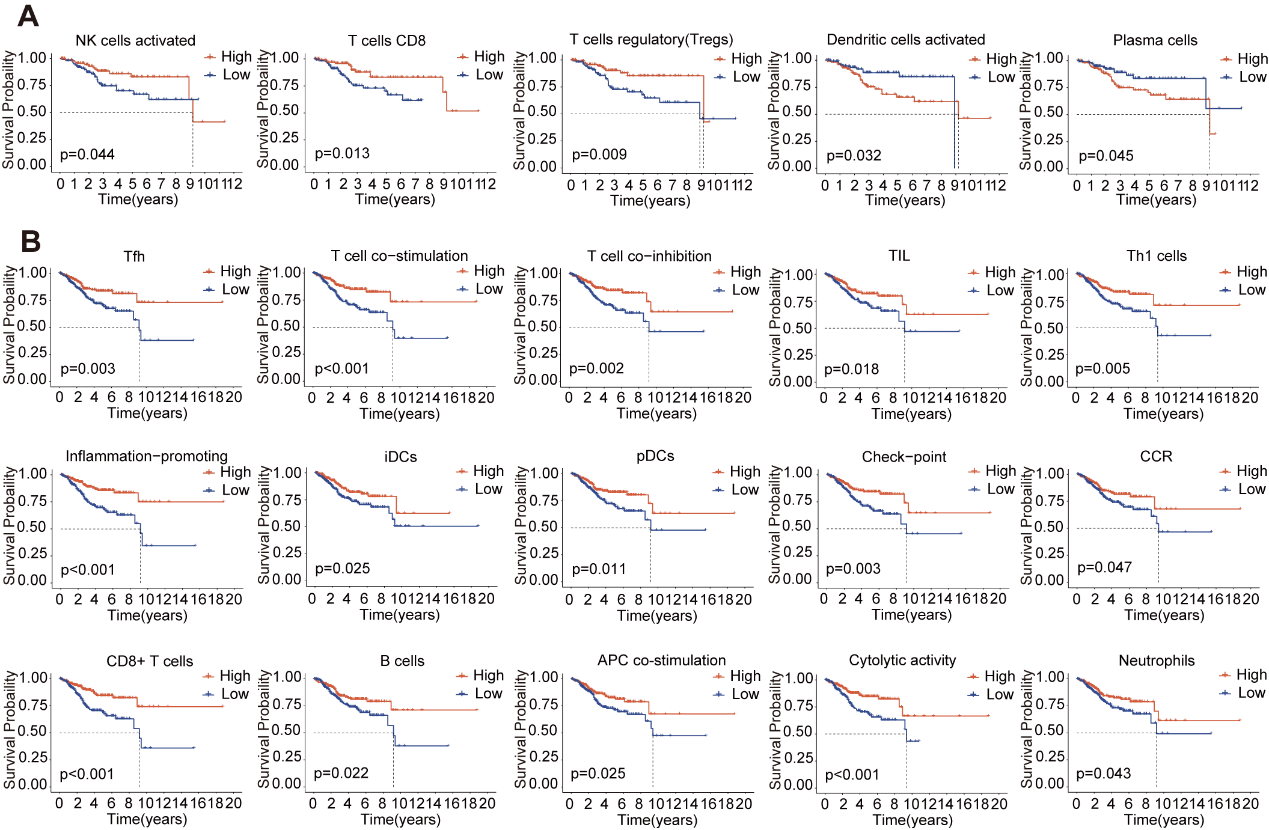
**

**Additional file 1. Fig. S1** Further survival analysis linked with immune infiltration. **(A)** Kaplan-meier analysis of significantly different immune cells. **(B)** Kaplan-meier analysis of significantly different immune pathways.
